# Effect of the Otago Exercise Program and Resonance Frequency Breathing in Patients Following Coronary Artery Bypass Grafting

**DOI:** 10.7759/cureus.115400

**Published:** 2026-08-29

**Authors:** Pranoti R Patil, Poovishnu T Devi

**Affiliations:** 1 Physiotherapy, Krishna College of Physiotherapy, Krishna Vishwa Vidyapeeth (Deemed to be University), Karad, IND; 2 Cardiopulmonary Sciences, Krishna College of Physiotherapy, Krishna Vishwa Vidyapeeth (Deemed to be University), Karad, IND

**Keywords:** autonomic nervous system, balance training, cardiac rehabilitation, coronary artery bypass grafting, exercise therapy, functional mobility, heart rate variability, otago exercise program, postoperative recovery, resonance frequency breathing

## Abstract

Background

Patients undergoing coronary artery bypass grafting (CABG) commonly experience reduced lower-limb strength, impaired balance, decreased functional mobility, and autonomic dysfunction during the postoperative period. These impairments may increase fall risk and limit daily activities. The Otago Exercise Program (OEP) is an evidence-based exercise intervention designed to improve lower-limb strength, balance, and functional performance, while resonance frequency breathing (RFB) has been shown to enhance cardiac autonomic regulation and heart rate variability (HRV). Although both interventions have demonstrated individual benefits, evidence regarding their combined application in phase II cardiac rehabilitation following CABG remains limited. Therefore, this study aimed to evaluate the effects of a combined OEP and RFB intervention on balance, lower-limb strength, functional mobility, and cardiac autonomic function in post-CABG patients.

Objective

To evaluate the effects of a combined OEP and RFB intervention on balance, lower-limb strength, functional mobility, and cardiac autonomic regulation as evaluated via HRV in patients undergoing phase II cardiac rehabilitation following CABG.

Materials and methods

A single-group pre-test and post-test experimental study was conducted among 40 post-CABG patients aged 40 to 60 years. The eight-week intervention combined supervised OEP multi-component exercises three times per week with daily home-based RFB paced precisely at six breaths per minute for 15 to 20 minutes. Lower extremity functional strength was evaluated by the 30-Second Chair Stand Test, and dynamic mobility was monitored with the Timed Up and Go (TUG) test. Static postural stability was evaluated using the Four-Stage Balance Test. Cardiac autonomic regulation was evaluated using short-term five-minute electrocardiographic (ECG) time-domain metrics, specifically tracked through the mean R-R interval and the root mean square of successive differences (RMSSD).

Results

Improvements were observed following the intervention. The TUG test results improved from 17.95 ± 2.86 seconds to 14.68 ± 2.93 seconds (p < 0.001), indicating improvement in functional mobility. The lower limb strength also improved. The 30-Second Chair Stand Test scores went up from 13.03 ± 1.83 to 15.78 ± 2.03 repetitions (p < 0.001). We also saw improvements in balance, tandem stance, and single-leg stance (p < 0.0001). Autonomic function also improved, with an increase in RMSSD from 18.03 ± 2.59 ms to 21.96 ± 2.59 ms (p < 0.001); these findings suggest improved parasympathetic activity.

Conclusions

The combined OEP and RFB intervention may support physical function, balance, functional mobility, and autonomic regulation in patients undergoing phase II cardiac rehabilitation after CABG surgery. The intervention appeared to be safe and useful in a home-based rehabilitation setting and may be a helpful approach in cardiac rehabilitation. However, the absence of a control group and separate OEP and RFB groups limits the ability to determine their individual or combined effects. Future studies with appropriate control and separate intervention groups are needed to confirm these findings.

## Introduction

On a global scale, cardiovascular diseases constitute a major cause of death and morbidity, accounting for 31% of all deaths globally, with coronary artery disease being the most prevalent form [1,2]. When medications and lifestyle modifications fail, patients frequently need coronary artery bypass graft (CABG) surgery as a definitive treatment option [3]. Patients undergoing CABG, mainly through great saphenous vein (GSV) graft harvesting, are at a significantly increased risk of falls. Prior studies show that approximately 38% of patients demonstrate an elevated risk of falling at baseline. Within 180 days of diagnosis, this vulnerability increases in nearly 60% of individuals and is correlated with higher rates of hospital readmission and mortality [4]. Furthermore, more than half of the individuals undergoing vascular surgery experience postoperative physical limitations, making them more vulnerable to falls during recovery [5].

Following CABG surgery, patients often become less physically active, compromising their ability to perform activities of daily living independently. This decline is commonly associated with structural and muscular impairments, including generalized muscle weakness, poor posture, fatigue of antigravity muscles, and altered body alignment. Balance impairment is an under-recognized consequence of CABG surgery and contributes to increased fall risk during postoperative recovery [6]. However, postural stability, particularly dynamic and functional balance, remains largely overlooked in the rehabilitation of CABG patients, emphasizing the need for rehabilitation programs to address these conditions and deficits [6,7]. Persistent discomfort, especially around the saphenous vein donor site in the leg, further contributes to postural instability and impaired balance during recovery [8]. Deficits in postural control are directly attributed to an increased frequency of falls, higher morbidity and mortality, and a greater financial burden on healthcare systems [9]. Harvesting of the GSV for CABG requires a lower-limb incision that typically begins approximately 5 cm above the medial malleolus and extends proximally. This surgical wound exposes patients to localized complications such as infections, hematomas, wound dehiscence, tissue necrosis, and altered nerve sensations [10,11]. Persistent pain and sensory loss at the graft harvesting site may impair proprioceptive feedback, thereby negatively affecting postural stability and coordination, which is directly associated with an increased risk of falls after the CABG [12,13]. In addition, postural deviations and muscular asymmetry frequently discourage patients from engaging in physical activity, which progressively deteriorates motor coordination and increases postural instability [14].

Heart rate variability (HRV) is an important physiological marker that provides insight into autonomic nervous system regulation of cardiac function [15]. HRV assesses the variation in time intervals between consecutive heartbeats using both time-domain and frequency-domain parameters. Standard clinical assessments commonly utilize measures such as the mean RR interval and the standard deviation of normal-to-normal intervals (SDNN) to evaluate these variations [15,16]. Previous studies have shown that CABG surgery results in a substantial reduction in HRV, often more than that seen following myocardial infarction. This reduction may occur due to several surgical factors, including mechanical manipulation of the heart, anesthesia, cardioplegia, and the use of cardiopulmonary bypass [17]. Reduced HRV following CABG indicates autonomic dysregulation and decreased cardiovascular adaptability [18].

The Otago Exercise Program (OEP) is a well-established home-based exercise program designed to improve lower extremity strength, enhance postural stability, and reduce the number of falls among older adults. This structured program includes lower extremity strengthening exercises, progressive walking exercises, and balance retraining activities, performed under supervision, making it a potentially useful intervention for postoperative deconditioning [18,19]. Clinical evidence suggests that OEP reduces fall risk by approximately 35% while improving postural stability, gait performance, and functional mobility. In addition, the program promotes greater physical independence, maintenance of functional capacity, and improves overall quality of life [20-24].

Resonance frequency breathing (RFB) is a paced breathing technique performed at approximately 4.5 to 7 breaths per minute to improve autonomic function [25]. This mechanism is based on respiratory sinus arrhythmia, in which heart rate increases during inspiration and decreases during exhalation. RFB enhances vagal tone, reduces stress and blood pressure, and improves HRV following CABG [25,26]. According to the neurovisceral integration model, increased vagal activity is directly associated with enhanced affect regulation and cognitive processing, so it will also be helpful for overall rehabilitation [27,28].

Although existing literature has separately demonstrated the benefits of balance training and RFB, particularly in older adults and cardiac populations, limited evidence exists regarding their combined application in post-CABG rehabilitation. Along with that, balance impairment and autonomic dysfunction frequently coexist following CABG surgery; however, rehabilitation programs generally target these strategies separately rather than an integrated rehabilitation program despite their potential interrelationship. There are limited studies present which focuses on combined balance and autonomic modulation strategies during the postoperative cardiac rehabilitation period following CABG surgery. Therefore, investigating a combined intervention targeting both functional and autonomic recovery may provide a more comprehensive rehabilitation approach for post-CABG patients. Therefore, the present study aims to evaluate the effectiveness of these interventions in improving functional outcomes and autonomic regulation in post-CABG patients.

## Materials and methods

Study design

This experimental study employed a single-group pre-test and post-test design to evaluate the effects of a combined OEP and RFB intervention on balance, functional mobility, lower-limb strength, and cardiac autonomic regulation in patients undergoing phase II cardiac rehabilitation following CABG. The study was conducted as part of a postgraduate research project after obtaining approval from the Institutional Ethics Committee. Written informed consent was obtained from all participants prior to enrolment.

Sample size calculation

Sample size estimation was performed using G*Power software (Heinrich Heine University Düsseldorf, Düsseldorf, Germany) based on pilot data obtained from preliminary observations. Assuming a significance level (α) of 0.05, statistical power of 80%, and a moderate expected effect size (d = 0.5), the minimum required sample size was calculated before participant recruitment. A total of 40 participants were recruited using convenience sampling.

Participants

The participants taking part in this study were adults aged 40 to 60 years who had undergone elective CABG using GSV grafting and were hemodynamically stable four to six weeks after surgery. Participants were required to understand and follow exercise instructions and breathing techniques with a caregiver at home. Exclusion criteria included active wound infection, severe lower-limb edema, deep vein thrombosis, uncontrolled arrhythmias, uncontrolled hypertension, significant respiratory disorders, neurological disorders affecting balance, musculoskeletal conditions limiting exercise participation, and preoperative dependence on walking aids.

Outcome measures

Electrocardiography Assessment

Cardiac autonomic function was evaluated using short-term HRV analysis. Electrocardiographic recordings were obtained using a single-channel ECG system with a sampling frequency of 500 Hz under standardized laboratory conditions. ECG recordings were collected in the morning between 8:00 AM and 10:00 AM, at least 24 hours after the last exercise session, to avoid acute post-exercise autonomic changes. Participants rested in a relaxed supine position for 10 minutes before data collection. A continuous five-minute ECG recording was then obtained during spontaneous breathing without respiratory pacing. Surface electrodes were applied according to standard ECG placement procedures. R peaks were automatically detected, and RR intervals were extracted for HRV analysis. All recordings were visually inspected for artifacts and ectopic beats. ECG recordings containing more than 5% artifacts were excluded from analysis. HRV analysis was performed using Kubios HRV software (Kubios Oy, Kuopio, Finland). The primary HRV parameters analyzed were mean RR interval (ms) and root mean square of successive differences (RMSSD; ms) [16].

RMSSD was calculated using the following formula:​​

 $\mathrm{RMSSD} = \sqrt{ \frac{1}{N-1} \sum_{i=1}^{N-1} \left(RR_{i+1}-RR_i\right)^2 }$

Where N = total number of R-R intervals; RR = R-R interval; RRi = duration of the ith R-R interval; RRi+1 = duration of the (i+1)th R-R interval; RRi+1 − RRi = difference between two consecutive R-R intervals.

Four-Stage Balance Test

Static balance and postural stability were assessed using the Four-Stage Balance Test. Participants were instructed to maintain four progressively challenging standing positions, including side-by-side stance, semi-tandem stance, tandem stance, and single-leg stance, for up to 10 seconds each. Performance was recorded according to the maximum duration achieved in each position. Data for stage 1 and stage 2 were omitted from statistical analysis because all participants were able to perform both stage 1 and stage 2 pre-intervention and post-intervention, respectively [29].

Timed Up and Go Test

The Timed Up and Go (TUG) test was used to evaluate functional mobility and dynamic balance. For the TUG evaluation, patients initiated the movement from a standard, armless chair, walked a taped three-meter course at their regular pace, turned, and returned to a seated posture. The total time required by the patient to complete the whole task was recorded accordingly [30].

Thirty-Second Chair Stand Test

Lower-extremity functional strength and endurance were evaluated using the 30-Second Chair Stand Test. Participants performed repeated sit-to-stand movements from a standard armless chair with their arms crossed over the chest. The total number of repetitions completed within 30 seconds was recorded [31].

Statistical analysis

The collected data were statistically analyzed using GraphPad InStat software (GraphPad Software Inc., San Diego, CA). Descriptive statistics were calculated and presented as mean ± standard deviation (SD) for continuous variables. The normality of the data was evaluated using the Shapiro-Wilk test. For normally distributed variables, pre- and post-intervention comparisons were performed using the paired t-test. For variables that did not follow a normal distribution, the Wilcoxon signed-rank test was used. A p-value < 0.05 was considered statistically significant. Cohen's d was calculated to estimate effect size.

Procedure

Participants were enrolled from a cardiac rehabilitation follow-up service and included adults aged 40-60 years who were hemodynamically stable four to six weeks after elective CABG with GSV grafting. This study was approved by the Institutional Ethics Committee of Krishna Vishwa Vidyapeeth (Deemed to be University), Karad, Maharashtra, India (Protocol No. 403/2025-2026), and informed consent was obtained from all participants. Baseline assessments included HRV analysis from a five-minute resting ECG, along with the 30 Second Chair Stand Test, TUG test, and Four-Stage Balance Test. The experimental OEP and RFB served as the primary rehabilitation protocol. Participants did not receive any other standard phase II cardiac rehabilitation pathways, such as hospital-based treadmill walking or cycling exercises, during the study period. Participants completed an eight-week intervention comprising supervised OEP sessions three times weekly and daily home-based RFB (15-20 minutes). Adherence was monitored through exercise logbooks and weekly follow-up calls, with a mean adherence rate of 92% and a 0% dropout rate. Post-intervention assessments were performed using the same procedures, and data were analyzed statistically. No major cardiovascular or musculoskeletal adverse events were reported during the study period. A flow diagram summarizing participant enrolment, intervention, and outcome assessment is presented in Figure 1.

**Figure 1:**
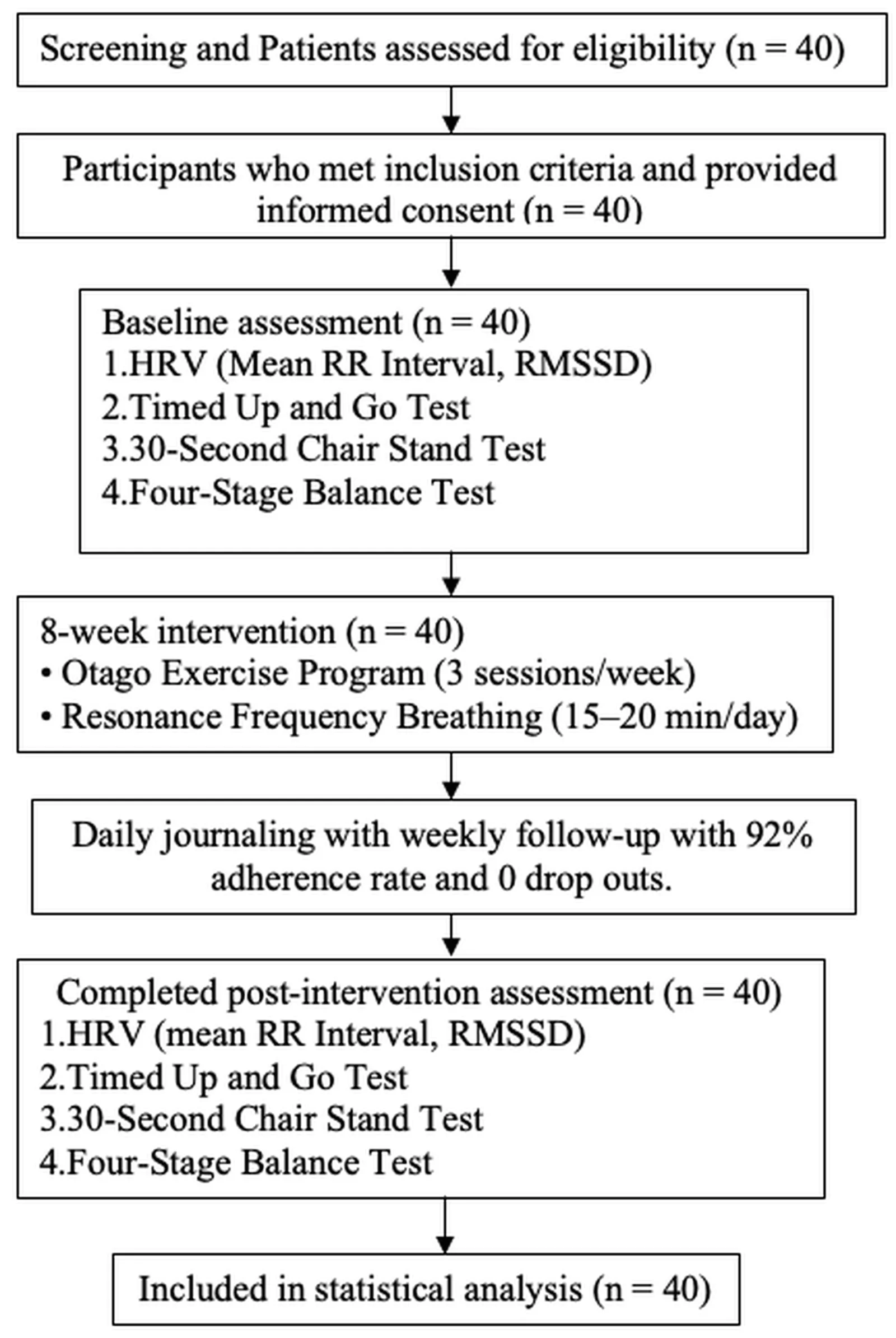
Flow diagram of participant screening, baseline assessments, eight-week multi-component intervention, and statistical analysis. n: number of participants; HRV: heart rate variability; RMSSD: root mean square of successive differences; RR interval: interval between consecutive R waves on the electrocardiogram.

## Results

The study included 40 post-CABG participants, comprising 24 (60.0%) males and 16 (40.0%) females, with an average participant age of 53.03 ± 5.56 years. Male participants were generally taller and heavier than female participants. Based on body mass index (BMI) classification, 20 (50.0%) participants were overweight, five (12.5%) were classified as obesity class I, 11 (27.5%) had normal weight, and four (10.0%) participants were underweight, as shown in Table 1.

**Table 1:** Baseline clinical demographics and weight status classification of the study population. Data are presented as mean ± standard deviation (SD) or as the number of participants (n) and percentages (%). Height is expressed in centimeters (cm), weight in kilograms (kg), and body mass index (BMI) was calculated by dividing body weight in kilograms by the square of height in meters and is expressed as kg/m².

Parameter	Total cohort (N = 40)	Male (n = 24)	Female (n = 16)
Age (years), Mean ± SD	53.03 ± 5.56	53.21 ± 5.59	52.75 ± 5.67
Height (cm), Mean ± SD	154.55 ± 8.27	156.40 ± 8.16	151.78 ± 7.91
Weight (kg), Mean ± SD	60.59 ± 11.51	63.39 ± 10.32	56.39 ± 12.24
BMI (kg/m²), Mean ± SD	25.13 ± 4.30	25.75 ± 3.73	24.20 ± 5.01
Underweight, n (%)	4 (10.0%)	1 (4.2%)	3 (18.8%)
Normal weight, n (%)	11 (27.5%)	6 (25.0%)	5 (31.2%)
Overweight, n (%)	20 (50.0%)	14 (58.3%)	6 (37.5%)
Obesity class 1, n (%)	5 (12.5%)	3 (12.5%)	2 (12.5%)

The study cohort was characterized by a high level of education and strong family support, with 28 (70%) participants having higher education and 35 (87.5%) participants living with a caregiver or family member. A total of 30 (75.0%) participants resided in suburban areas, as shown in Table 2.

**Table 2:** Baseline socioeconomic characteristics of the study population.

Socioeconomic characteristics	Frequency (n)	(%)
Education level		
Primary/secondary	12	30.00%
Higher education	28	70.00%
Living arrangement		
Living with caregiver/family	35	87.50%
Living alone	5	12.50%
Geographical setting		
Sub-urban	30	75.00%
Rural	10	25.00%

Hypertension was present in 28 (70%) participants, and type 2 diabetes mellitus was present in 13 (32.5%) participants; these were the most common comorbidities. Chronic obstructive pulmonary disease was present in three (7.5%) and acute kidney disease in two (5%) participants. All participants received antiplatelet therapy, while the majority were prescribed statins and beta blockers, as shown in Table 3.

**Table 3:** Prevalence of baseline comorbidities and pharmacological therapy profiles in the study population. Data are presented as the number of participants (n) and percentages (%). ACE: angiotensin-converting enzyme; ARBs: angiotensin II receptor blockers.

Comorbidities	Number of participants (n)	%
Hypertension	28	70%
Diabetes mellitus (type 2)	13	32.50%
Chronic obstructive pulmonary disease	3	7.50%
Acute kidney disease	2	5.00%
Pharmacological therapy		
Antiplatelet therapy	40	100%
Beta-blockers	33	82.50%
Statins (lipid-lowering)	36	90%
ACE inhibitors/ARBs	25	62.50%

The baseline data showed poor physical strength and reduced heart autonomic function among participants. Most required support during the 30-Second Chair Stand (33, 82.5%) and TUG (34, 85.0%) tests, indicating limited mobility. Balance performance also declined sharply, with standing time decreasing from 8.07 seconds in tandem stance to 0.40 seconds in single-leg stance. The low RMSSD value (18.03 ms) further suggests reduced parasympathetic activity, as shown in Table 4.

**Table 4:** Baseline functional mobility, postural balance, and heart rate variability parameters of the study cohort. Data are presented as mean ± standard deviation (SD). Thirty-Second Chair Stand Test values are expressed as the number of repetitions (reps); Timed Up and Go Test and Four-Stage Balance Test values are expressed in seconds (s); mean RR interval and RMSSD are expressed in milliseconds (ms). RMSSD: root mean square of successive differences; RR interval: interval between consecutive R waves on the electrocardiogram.

Variable	Mean ± SD	Minimum - Maximum	Support required (n) (%)
30-Second Chair Stand Test (repetitions)	13.03 ± 1.83	10 - 16	33 (82.5%)
Timed Up and Go Test (seconds)	17.95 ± 2.86	13 - 25	34 (85.0%)
Four-Stage Balance Test, Stage 3 (seconds)	8.07 ± 2.37	3 - 10	-
Four-Stage Balance Test, Stage 4 (seconds)	0.40 ± 1.15	0 - 5	-
Mean RR interval (ms)	732.35 ± 33.35	671.4 - 804.8	-
RMSSD (ms)	18.03 ± 2.59	12.2 - 25.4	-

The intervention led to noticeable improvements in mobility, balance, and heart autonomic function. Participants performed better in the 30-Second Chair Stand Test, completing 2.75 more repetitions, and completed the TUG test 3.27 seconds faster. The need for external support also decreased from over 33 (82%) to less than 12 (33%). Balance improved, with longer hold times in single-leg stance. In addition, heart function showed associated improvement with intervention, as reflected by increases in mean RR interval (+23.55 ms) and RMSSD (+3.93 ms), indicating better parasympathetic activity, as shown in Table 5.

**Table 5:** Pre- and post-intervention outcomes: Effect of combined Otago exercises and resonance frequency breathing on physical performance and autonomic function (N = 40). RMSSD: root mean square of successive differences; RR interval: time interval between consecutive R waves on the electrocardiogram.

Outcome measure	Pre-intervention, Mean ± SD	Post-intervention, Mean ± SD	Mean difference	Support rate, Pre, n(%)	Support rate, Post, n (%)
30-Second Chair Stand Test (repetitions)	13.03 ± 1.83	15.78 ± 2.03	+2.75	33 (82.5%)	12 (30.0%)
Timed Up and Go Test (seconds)	17.95 ± 2.86	14.68 ± 2.93	−3.27	34 (85.0%)	13 (32.5%)
Four-Stage Balance Test Stage 3 (seconds)	8.07 ± 2.37	9.15 ± 1.55	+1.08	-	-
Four-Stage Balance Test Stage 4 (seconds)	0.40 ± 1.15	3.85 ± 3.18	+3.45	-	-
Mean RR interval (ms)	732.35 ± 33.35	755.90 ± 33.00	+23.55	-	-
RMSSD (ms)	18.03 ± 2.59	21.96 ± 2.59	+3.93	-	-

Statistical analysis showed significant post-intervention improvements across parameters (p < 0.05). The intervention had large effect sizes for the TUG (t = 12.71, d = 2.01) and 30-Second Chair Stand (t = 7.53, d = 1.19) tests. Postural stability improved in stage 3 (W = 20, Z = -3.22, p = 0.0013, d = 0.63) and stage 4 (W = 9, Z = -4.53, p = 0.000006, d = 1.11) using non-parametric analysis. Autonomic parameters also improved for mean RR interval (t = 9.07, d = 1.43) and RMSSD (t = 8.45, d = 1.34), as shown in Table 6.

**Table 6:** Paired-samples t-test and Wilcoxon signed-rank test outcomes and Cohen's d effect size calculations for physical and autonomic parameters (N = 40). We performed statistical analysis using the paired Student's t-test to compare pre- and post-intervention values for normally distributed parameters. For parameters that did not meet normality assumptions due to floor effects and non-normal distributions, specifically the Four-Stage Balance Test, stages 3 and 4, we used the non-parametric Wilcoxon signed-rank test to evaluate statistical significance. A p-value less than 0.05 was considered statistically significant. Cohen's d was calculated across all metrics to estimate a standardized effect size for comparison. W: Wilcoxon signed-rank test statistic; Z: standardized normal approximation score; t: Student's paired t-test statistic; p: probability value; d: Cohen's effect size index; RMSSD: root mean square of successive differences; RR interval: time interval between consecutive R waves on an electrocardiogram.

Outcome measure	Test statistics	p-value	Standard Z score	Effect size (Cohen’s d)	Result
Four-Stage Balance Test – Stage 3	W = 20	0.0013	-3.22	0.63	Statistically significant
Four-Stage Balance Test – Stage 4	W = 9	0.000006	-4.53	1.11	Statistically significant
30-Second Chair Stand Test	t = 7.53	<0.001	-	1.19	Statistically significant
Timed Up and Go Test	t = 12.71	<0.001	-	2.01	Statistically significant
Mean RR Interval	t = 9.07	<0.001	-	1.43	Statistically significant
RMSSD	t = 8.45	<0.001	-	1.34	Statistically significant

## Discussion

The present study demonstrated significant improvements in lower-limb strength, functional mobility, balance, and autonomic regulation following the eight-week protocol. These findings suggest that addressing both physical and autonomic impairments at the same time may help with recovery during phase II cardiac rehabilitation. Noticeable improvements were observed across outcome measures. The improvements observed in mobility, balance, and HRV indicate that combining OEP with RFB may address both physical and autonomic impairments commonly seen after CABG.

Lower-limb strength improved following the intervention, as demonstrated by the increase in 30-sec chair stand repetitions from 13.03 ± 1.83 to 15.78 ± 2.03 repetitions, representing an improvement of 18.22%. This improvement may be attributed to the progressive strengthening of lower extremity musculature, which results in improvement in sit-to-stand ability, gait efficiency, and independence in daily activities. This finding is consistent with an Otago case report by Sari et al. (2026) [32], who demonstrated that balance exercise and the OEP were associated with a significant improvement in functional mobility among a community of older adults, as indicated by reductions in the TUG test following the intervention. Rathi et al. (2022) [33] reported a 2.73-second improvement, and our study reported a 3.27-second improvement on the TUG test.

Studies by Chiu et al. (2021) [34] and Wang et al. 2025 [35] reported gains in lower extremity strength, functional independence, and physical performance after Otago-based training. The improvement observed in the present study may be attributed to the progressive strengthening component of the OEP, which targets muscle groups involved in sit-to-stand activities, gait, and postural control. Furthermore, the reduction in support requirement from 82.5% to 30% indicates that the intervention produced clinically meaningful improvements in functional independence.

Functional mobility also improved significantly, with TUG performance decreasing from 17.95 ± 2.86 seconds to 14.68 ± 2.93 seconds, corresponding to an 18.22% improvement. Improved mobility may link with increase in lower-limb strength and postural control, which help participants to perform ambulatory tasks more efficiently with reduced fear of falling. The Thirty-Second Chair Stand Test performance improved by 2.75 repetitions in the present study. This improvement was greater than the 1.07-repetition increase reported by Rathi et al. (2022) [33], who observed significant mobility improvements following the implementation of the OEP. These results suggest that meaningful mobility gains can be achieved within a relatively short period following CABG surgery. Improved TUG performance may be explained by enhanced lower-limb strength, improved balance, better neuromuscular co-ordination, and increased confidence during ambulation. Additionally, the reduction in support requirement during TUG from 85% to 32.5% highlights the practical significance of these results and suggests improved functional mobility during daily activities.

Tandem stance performance increased from 8.07 ± 2.37 seconds to 9.15 ± 1.55 seconds, while single-leg stance performance improved markedly from 0.40 ± 1.15 seconds to 3.85 ± 3.18 seconds. These findings are consistent with the work of Khumpaneid et al. (2022) [36], Chiu et al. 2021 [34], Martins et al. (2018) [24], and Wang et al. (2025) [35], all of whom reported significant improvements in static and dynamic balance following participation in Otago-based exercise programs. Similarly, the pioneering studies by Campbell and Robertson [19] demonstrated reductions in fall risk and improvements in postural stability following implementation of the OEP in older adults. The substantial improvement observed in single-leg stance performance is particularly noteworthy because single-leg balance is considered a sensitive indicator of postural control and fall risk. The improvement in balance due to lower-limb strengthening, balance training, and gait re-education with specific progressive training may be particularly important for CABG patients experiencing balance disturbances and reduced physical activity.

An important finding of the present study was the improvement in cardiac autonomic function. Mean RR interval increased from 732.35 ± 33.35 ms to 755.90 ± 33.00 ms, while RMSSD increased from 18.03 ± 2.59 ms to 21.96 ± 2.59 ms, representing a 21.8% increase. RMSSD is a well-established marker of parasympathetic activity and cardiac vagal modulation. RFB may enhance parasympathetic modulation through synchronization of cardiovascular and respiratory rhythms. This mechanism increases baroreflex sensitivity and helps to improve HRV parameters such as RMSSD. Reduced HRV is frequently reported following CABG surgery and has been associated with adverse cardiovascular outcomes, delayed recovery, and increased mortality risk. Therefore, the increase in RMSSD observed in the present study suggests a favorable autonomic adaptation following the intervention. Furthermore, systematic reviews have consistently shown that structured rehabilitation programs can enhance parasympathetic activity and improve cardiovascular regulation following cardiac surgery [37].

The observed autonomic improvements may be partly explained by the inclusion of RFB. Slow-paced breathing performed near an individual's resonance frequency has been shown to increase baroreflex sensitivity, optimize autonomic balance, and enhance vagal activity. These outcomes are consistent with the work of Lehrer et al. (2000) [28] and Gitler et al. (2025) [38], who reported improvements in HRV indices, including RMSSD, following RFB and HRV biofeedback interventions. Unlike conventional rehabilitation programs that primarily target physical recovery, RFB directly influences autonomic nervous system regulation. Consequently, the combination of OEP and RFB may have produced effects, with OEP improving physical performance and balance while RFB enhanced autonomic function and cardiovascular regulation. The effect sizes observed in the present study further support the clinical significance of the intervention. The 30-Second Chair Stand Test demonstrated a large effect size (d = 1.19), while TUG showed a larger effect size (d = 2.01). Similarly, stage 4 balance performance (d = 1.06), mean RR interval (d = 1.43), and RMSSD (d = 1.34) all demonstrated large effects. These results suggest that the intervention had a substantial impact on functional and physiological recovery among post-CABG patients.

The outcomes of the present study are further supported by systematic reviews conducted by Dibben et al. (2023) [39], which demonstrated that cardiac rehabilitation following CABG surgery helps to benefit coronary heart disease patients by improving cardiovascular outcomes; in addition, it is cost-effective, as it is an exercise-based cardiac rehabilitation. Unlike many previous studies that focused solely on aerobic exercise, resistance training, or conventional cardiac rehabilitation, the present study integrated a balance-oriented exercise program with autonomic modulation through RFB. This integrated approach may explain the simultaneous improvements observed across mobility, balance, strength, and HRV outcomes.

The study utilized a single-group pre-test and post-test design without a control group, limiting the ability to establish direct relationships between the intervention and observed improvements. The lack of a control group prevents differentiation between the intervention effects and natural postoperative recovery. The relatively small sample size and convenience sampling method may further restrict the validity of the findings. HRV analysis was limited to basic time-domain parameters because of equipment limitations, and advanced frequency-domain analysis could not be performed. Frequency-domain HRV parameters could not be evaluated because of equipment limitations. The main limitations of this home-based protocol are the lack of direct clinical supervision and the reliance on patient self-reporting biases. The study also lacked long-term follow-up assessment; therefore, the sustainability of improvements remains unknown. Future studies should include larger sample sizes and randomized controlled trials with control-group designs to establish stronger evidence regarding the effectiveness of the combined OEP and RFB intervention in post-CABG rehabilitation.

Overall, the outcomes of the present study demonstrate that an eight-week program combining the OEP and RFB can significantly improve lower-limb strength, functional mobility, balance performance, and cardiac autonomic function in post-CABG patients. These results support the feasibility and potential clinical value of incorporating OEP and RFB within phase II cardiac rehabilitation programs and suggest that this combined approach may facilitate both functional recovery and cardiovascular autonomic restoration following CABG surgery.

## Conclusions

The combined OEP and RFB intervention may support physical function, balance, functional mobility, and autonomic regulation in patients undergoing phase II cardiac rehabilitation after CABG surgery. The intervention appeared to be safe and useful in a home-based rehabilitation setting and may be a helpful approach in cardiac rehabilitation. However, the absence of a control group and separate OEP and RFB groups limits the ability to determine their individual or combined effects. Future studies with appropriate control and separate intervention groups are needed to confirm these findings.
